# An Easily Accessible Web-Based Minimization Random Allocation System for Clinical Trials

**DOI:** 10.2196/jmir.2392

**Published:** 2013-07-19

**Authors:** Lan Xiao, Qiwen Huang, Veronica Yank, Jun Ma

**Affiliations:** ^1^Research InstitutePalo Alto Medical FoundationPalo Alto, CAUnited States; ^2^School of MedicineStanford UniversityStanford, CAUnited States

**Keywords:** randomized controlled trials, randomization, minimization, adaptive randomization, Kullback–Leibler divergence, Web-based

## Abstract

**Background:**

Minimization as an adaptive allocation technique has been recommended in the literature for use in randomized clinical trials. However, it remains uncommonly used due in part to a lack of easily accessible implementation tools.

**Objective:**

To provide clinical trialists with a robust, flexible, and readily accessible tool for implementing covariate-adaptive biased-coin randomization.

**Methods:**

We developed a Web-based random allocation system, MinimRan, that applies Pocock–Simon (for trials with 2 or more arms) and 2-way (currently limited to 2-arm trials) minimization methods for trials using only categorical prognostic factors or the symmetric Kullback–Leibler divergence minimization method for trials (currently limited to 2-arm trials) using continuous prognostic factors with or without categorical factors, in covariate-adaptive biased-coin randomization.

**Results:**

In this paper, we describe the system’s essential statistical and computer programming features and provide as an example the randomization results generated by it in a recently completed trial. The system can be used in single- and double-blind trials as well as single-center and multicenter trials.

**Conclusions:**

We expect the system to facilitate the translation of the 3 validated random allocation methods into broad, efficient clinical research practice.

## Introduction

Randomized controlled trials (RCTs) are the gold standard for assessing efficacy or effectiveness of biomedical and behavioral treatments. The ideal randomization procedure would achieve the following goals: (1) balanced arm sizes, (2) no selection bias (ie, unpredictability of future treatment assignment), and (3) no accidental bias (ie, low probability of confounding because of between-treatment imbalance in pretreatment characteristics of prognostic importance). However, no randomization procedure can achieve all these goals in every circumstance, which makes randomization conceptually straightforward but practically complex. Simple randomization—also called *unrestricted randomization*—minimizes selection bias but not accidental bias [1]. Hence, several restricted randomization procedures have been developed to address these limitations.

A practical solution that minimizes accidental bias when multiple prognostic factors are involved is the covariate-adaptive biased-coin randomization procedure widely known as the Pocock-Simon minimization method [2]. This method achieves marginal balance by accounting for all the selected pretreatment covariates for the previously assigned subjects and assigning the next subject to a treatment with a probability in favor of minimizing the overall imbalance across the covariates. Use of nonextreme allocation probabilities (eg, 2/3:1/3 in a 2-arm trial) helps protect unpredictability [2,3]. A 2-way minimization method is another way to protect unpredictability by using probability to minimize either the “imbalance in the total numbers of subjects” or the “imbalance in the distribution of prognostic factors” [4]. Both Pocock-Simon and 2-way minimization methods only allow for balancing by categorical prognostic factors. However, categorizing continuous covariates may not always be feasible or preferable (eg, because of a lack of scientific basis for or consensus on cut points). Endo et al [5] extended the Pocock-Simon approach to incorporate continuous prognostic factors in 2-arm trials by using the symmetric Kullback-Leibler divergence (KLD) (ie, Jeffrey’s divergence) index [6,7]. They demonstrated in a simulation study that, when continuous prognostic factors were included, the symmetric KLD method produced better covariate balance between treatments and more robust estimates of treatment effects than the Pocock-Simon method [5]. Despite their notable advantages and recommended use by many statistical and trialist commentators [8], these minimization methods remain infrequently used, to a large extent because of a lack of easily accessible tools [9].

In 2000, Kenjo et al [10] published their Web-based allocation system for multisite clinical trials using Pocock-Simon’s minimization method, but as noted in Cai et al [11], that system did not appear to support multiple trials simultaneously or address blinding. Cai et al [11] subsequently developed a Web-based allocation system also based on the Pocock-Simon method specifically for double-blind trials (see subsequent definition). Although there is a freely available online directory of randomization software [12], only 2 downloadable programs of those listed, Minim and MinmPy [13], support minimization methods. QMinim, an online version of MinmPy, is also freely available [14]. However, none of these minimization tools include role management function. In other words, they do not allow for the granting of different access privileges to different users and, therefore, cannot support double-blind trials. In addition, each only offers a single minimization method.

To promote increased use of minimization methods in various study designs and settings, we have developed a robust Web-based randomization system, named MinimRan, with flexible and user-friendly features, including (1) choice of the minimization method (Pocock-Simon, symmetric KLD, or 2-way minimization), (2) differentiated access privileges for efficient user–project role management within and across projects within research teams, (3) simultaneous system access by multiple users within and across multiple sites, (4) convenient graphical user interfaces (GUI) for information input and output, (5) proper protections of blinding in single- and double-blind trials, (6) standardized reports for continuous, timely quality monitoring of the randomization process, and (7) interactive tools for information updates and error corrections.

## Methods

### System Design

We designed this Web-based random allocation system to support sequential covariate-balanced assignment of subjects in single-site and multisite trials that use single- or double-blind designs. Blinding helps prevent the subjects and/or researchers from biasing the outcome of a study. The definitions of single- and double-blind designs are described in the Multimedia Appendix 1.

As noted previously, our system’s statistical algorithms are based on Pocock-Simon’s minimization method (for trials with 2 or more arms), Endo et al’s symmetric KLD minimization method [5] (currently limited to 2-arm trials), and 2-way minimization method (currently limited to 2-arm trials). All 3 methods can be applied to single-site or multisite studies. Users may create new projects and manage multiple existing projects, and may access comprehensive account management and monitoring functions—all within 1 account for the same research team.

### Three-Tier System Architecture

The system uses a 3-tier architecture, which is the most widely used browser–server architecture. The 3-tier architecture consists of a presentation tier, logic tier, and data tier (Multimedia Appendix 1). The presentation tier is the user interface, which collects and displays information from the logic tier through a Web browser. The logic tier uses Tomcat server as the Web server and the Java application Java Server Page (JSP) along with Cascading Style Sheets (CSS) and JavaScript to build the Web application. The data tier is the back-end MySQL database server. Java Database Connectivity (JDBC) achieves database-independent connectivity between the Java programming language and the MySQL database. The detailed technical description of how the 3 tiers work together is included in Multimedia Appendix 1.

The system can be accessed by using Internet Explorer 8.0 or higher or Firefox [15].

### User Roles

**Figure 1:**
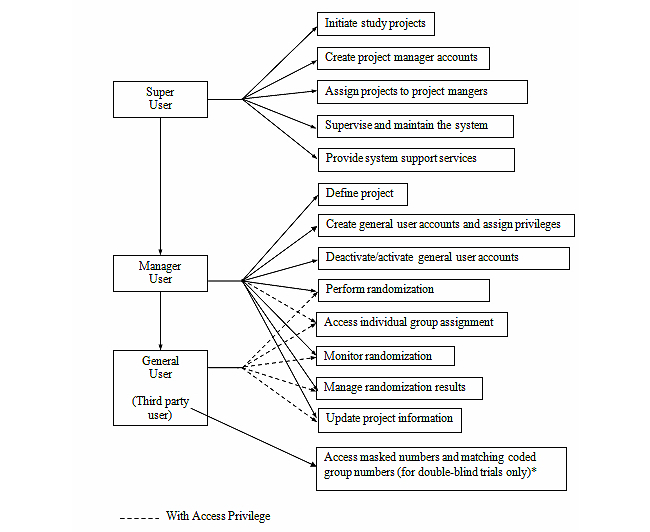
User roles.

Three types of users—super, project manager, and general—can access the system with different types of privileges (Figure 1). Our technical team retains the role of the super user (and serves as the system administrator). The privileges of this role include (1) initiating study projects, (2) creating project manager accounts, (3) assigning projects to new or existing project managers, (4) supervising and ensuring proper uses of the system, and (5) planning for and responding to service outages and other system problems. After the super user authorizes an account for the project manager on a research team, the manager can then carry out the following project-specific activities: (1) defining project characteristics (eg, single- or double-blind trial, number of study groups, study sites, prognostic factors), (2) creating general user accounts with individual privileges specified, (3) deactivating general user accounts that are no longer needed, (4) performing randomization, (5) monitoring randomization with the ability to view and verify randomized records as appropriate to manager’s blinding status (eg, only masked numbers available for double-blind trial), (6) managing randomization results (eg, generating summary tables, downloading allocated records), and (7) updating project information (eg, adding study sites). General users on a given project can perform randomization and 1 or more of the other functions previously listed according to each person’s privileges as assigned by the project manager. To help ensure blinding, the system will prompt the project manager to specify on a project-by-project basis which user(s) have permission to access the key that reveals subjects’ group assignments. In a single-blind trial, the project manager and/or 1 or more general users may be granted permission. In a double-blind trial, the key should be accessed and kept only by a third party and not given to any researchers involved in the study, including the project manager and general users performing randomization, until the study is over. When the project manager designates a general user as a third party with permission to access the group assignment key (for details, see section Randomization Process and Blinding), the system will automatically disable all randomization-related functions for that person.

### Creation and Maintenance of Research Projects

**Figure 2:**
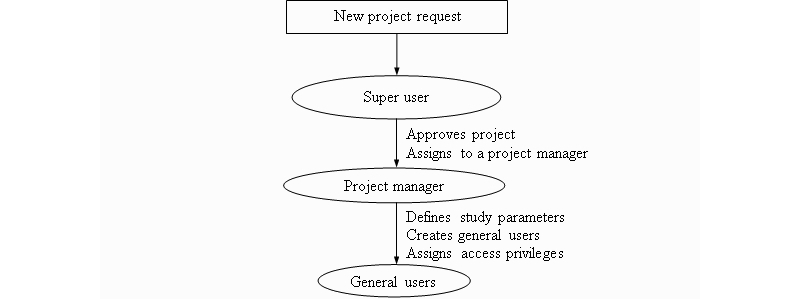
Steps to create a new project.

The steps for creating a new project are as follows (Figure 2):

1. Request to initiate a new project submitted by an existing or a new project manager. A brief description of the study must be provided that includes project name, purpose of study, beginning and expiration dates, funding source with grant number(s) if applicable, and applicant’s contact information.
2. The super user will create a new project using the information provided and assign it to the project manager’s account, which is also created at this point if there is not an existing account.
3. Definition of study parameters by the authorized project manager. The parameters include single- or double-blind trial, number of study groups and group names (optional), number and short names of study sites, projected maximum number of subjects for each study site (required for double-blind trials only), minimization method selected (Pocock-Simon, symmetric KLD, or 2-way minimization), biased assignment probability (not required for 2-way minimization), prognostic factors, and levels of each categorical factor. If the Pocock-Simon method is chosen, the user also needs to specify the number of initial subjects allocated using simple randomization (n=1 by default). The system recommends to users that they select simple randomization for the first 10 to 15 subjects as a strategy to prevent guessing of assignments when cases are few [16].
4. Creation of general user accounts and assignment of individual privileges by the project manager (for details, see section User Roles and Figure 1). General users are prompted to set their individual username and password when they log on for the first time.

Study projects can have 1 of 3 status designations: pending, ongoing, or expired. A project is pending when the authorized project manager has yet to complete steps 3 and 4 outlined previously. Once the setup is completed, randomization can then begin and the project’s status changes to ongoing, and will remain as such until the expiration date specified during the project initiation process (see step 1). Thirty days before a project’s expiration date, the system will generate an alert for the project manager who may at that point request an extension by emailing the super user. For expired projects, the project manager can view and download data records, but functions related to randomization of new subjects are deactivated. An expired project may be reactivated by the super user upon request.

### Randomization Process and Blinding

The system gives users the option of uploading records with subject IDs and prognostic factors for randomization by using a comma-separated values (CSV) data file or manually entering records 1 at a time. A CSV is a simple, widely supported file format in scientific, business, and consumer applications, and it permits efficient transfer of tabular data between programs. Within a given trial, both input methods are available for the user to select during each randomization run, and switching between methods from 1 run to the next is permitted. With both methods, data validation before randomization is strongly encouraged in all cases. Specifically, the system prompts the user to verify the inputted subject information before executing the randomization. The system also automatically checks the values of the prognostic factors entered each time against user-defined logic rules and generates an error message if any rule is violated. After data validation, the system opens the Pocock-Simon, symmetric KLD, or 2-way minimization method procedure depending on the user’s selection. The system automatically generates random numbers and outputs the randomization results using system-generated coded group numbers (eg, 1, 2, or 3 for a 3-arm study) or group names (if the manager user opts to describe group numbers) for single-blind trials or using masked individual numbers (ie, system-generated random numbers with a preceding *M*) for double-blind trials. For the former, only users with permission to access the key that identifies the subjects (subject IDs provided by the research team) and to which group they belong (coded group numbers or group names) can see the randomization results. For the latter, the system generates a Masked_Num table upon completion of the project initiation steps (section Creation and Maintenance of Research Projects) and before randomization of the first subject in a double-blind trial. The table contains masked numbers and matching coded group numbers or group names (by study site if a multisite trial), which only a designated third-party general user can access (section User Roles) and download (as a CSV file) for encoding the treatments (eg, using masked numbers on drug bottle labels for distribution and tracking). The system provides project managers and general users performing randomization on double-blind trials with subjects’ assigned masked numbers but not the associated group numbers. The user-projected maximum number of subjects to be enrolled plus 10% more determines the number of masked numbers generated by the system. The system will generate additional masked numbers if 90% of the initial set of numbers for any of the study group have been assigned. If the study includes multiple sites, this assignment will apply for each site. A designated user on a single-blind trial who is involved in conducting the research and has permission to access randomization results and the third party on a double-blind trial will be responsible for matching the randomization results and the actual study groups. As is standard practice in randomized clinical trials, this information should be kept in confidence (ie, not revealed to the researchers and participants who should remain blinded) until the study is ready to break the blind.

As previously mentioned, the system supports randomization at multiple sites and by multiple users. To prevent the race condition in a multi-user environment (ie, 2 or more users from the same study performing randomization tasks simultaneously), the system randomizes subjects in order of auto-incremented unique numbers that MySQL automatically generates when new records are inserted. The system also prevents duplicate randomization of the same subject ID within a project and will display an error message if this occurs. In spite of existing logical error checking provided by the system, some human entry errors may still be unavoidable. If the errors are found after randomization, the system only allows project managers to correct the entry errors and requires that he/she specify the reason, but the randomization results that happened before the corrections will remain unchanged. Randomization of any new subjects after the corrections, thus, will be based on the corrected information. The action of revision will be recorded and traceable in the randomization process data. Detailed randomization process data (eg, study ID, factor values, random number, random probability) are captured in the back-end database and are retrievable to permit quality control and replication. A manager user with permission to access group assignments can download randomization process data for current and expired single-blind trials that he/she manages. For double-blind trials, however, the data can be requested from the super user only if the manager user attests in writing that a trial has broken the blind.

### Back-End Database Design

The relational database built for MinimRan makes the system dynamic, flexible, scalable, and reliable The system uses MySQL to generate 8 tables for both single-blind and double-blind trials and 1 additional table for double-blinded trials only. The contents of each table and the relationships with other tables are described in detail in Multimedia Appendix 1.

### Statistical Methods

Minimization is designed to minimize marginal imbalance over multiple important prognostic factors as each consecutive treatment assignment is made. The treatment assignment that results in the least overall imbalance will be chosen with a high probability (*P*_i_), thereby increasing the chance of maximizing balance among the prognostic factors. The choice of *P*_i_ determines the degree of balance and the predictability of treatment assignment. Both Pocock-Simon and symmetric KLD methods define *P*_i_ as a fixed value throughout the whole or partial randomization stage, whereas 2-way minimization method defines dynamic *P*_i_ as a function of the imbalance in the total numbers of subjects. Depending on the type of prognostic factors chosen and user preference, in our system, users can choose one of these 3 methods for measuring imbalance.

#### Pocock-Simon’s Imbalance Score

**Figure 3:**
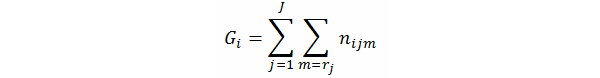
Equation for marginal treatment total.

The first option for measuring imbalance is to use the Pocock-Simon minimization method, which requires that continuous prognostic factors be categorized to calculate treatment imbalance [2,17]. At an arbitrary point in the succession of randomizations and after the specified number of initial subjects for whom simple randomization is used is met, denote n_ijm_ as the number of patients with level m of factor j who have been previously assigned to treatment arm i (j=1,2,...,J; m_j_= 1,2,...,M_j_; and i=1,2,..., I, where J, M_j_, and I are the numbers of prognostic factors, levels of factor j, and treatment arms, respectively). Let the next participant entering the trial have levels r_1_, r_2_,...,r_J_ on the prognostic factors 1,...,J. Pocock and Simon proposed several ways of measuring the cumulative imbalance on the previously assigned subjects and after assignment of a new participant [2,17]. We chose to balance the marginal treatment totals for each level of each patient factor in our system [17]. Figure 3 displays the equation used, where G_i_ is the marginal treatment total if the new participant is assigned to treatment i. The G scores corresponding to each treatment i are then ranked from the smallest to the largest and assigned with the corresponding *P*_i_.

#### The Symmetric KLD Index

**Figure 4:**
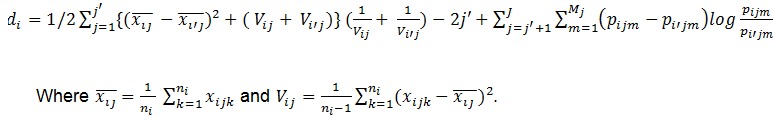
Equation for the KLD index.

The second option for measuring imbalance is to use the algorithm that measures the amount of imbalance between treatments (currently limited to 2) over multiple prognostic factors by computing a symmetric KLD index after a permuted block of the first 4 subjects have been assigned [5]. Let treatment be coded i (i=1,2). Consider any arbitrary point with the number of subjects n>4. Let x_ijk_ be the value of kth (k=1,2,...,n_i_) participant assigned to treatment i with the jth (j=1,2,...,j′) continuous prognostic factor, and p_ijm_ be the proportion of subjects assigned to the level m (m=1,2,...,M) of the jth (j+1,...,J) categorical prognostic factor. The difference in the distribution of prognostic factors between 2 treatments i and i′ (d_i_) can be measured as shown in the equation in Figure 4.

When the new participant n+1 is enrolled, d_i_ is calculated by assuming that this individual is allocated to i where i can be either treatment. Hence, the total number of subjects for treatment i becomes n_i_ +1 and the number of subjects for the other treatment i′ (n_i_′) remains unchanged. The value d_i_ represents the amount of imbalance in treatment i assuming the new subject is allocated to this treatment. The higher probability *P*_i_ is then assigned to the treatment arm with lower d_i_. The symmetric KLD algorithm assumes a multivariate normal distribution for continuous prognostic factors, although Endo et al [5] demonstrated that the algorithm was robust to nonnormally distributed data. If the symmetric KLD method is chosen, the system displays a message to alert the user to the multivariate normal distribution assumption and advises consulting a biostatistician on the need for data transformation if it is believed that serious violations may occur given prior knowledge of the expected distributions of the continuous factors used in the trial.

#### Two-Way Minimization Method

This method (currently limited to trials with 2 arms) calculates the imbalance in the total numbers of subjects and the imbalance in the distributions of prognostic factors. It then chooses, based on the defined probability *P*_i_, to minimize either 1 of these 2 imbalances.

Consider an arbitrary point in the trial after a simple randomization scheme allocates at least 1 subject in each group. Let n_T_ and n_C_ denote the total numbers of subjects allocated to the treatment group and the control group.

**Figure 5:**
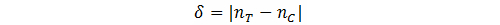
Equation for imbalance in the total numbers of subjects for two-way minimization method.

**Figure 6:**
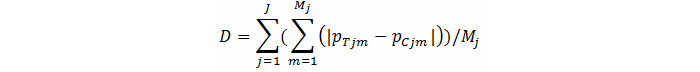
Equation for imbalance in the distributions of prognostic factors for two-way minimization method.

For the equation used for imbalance in the total numbers of subjects, see Figure 5. For the equation used for imbalance in the distributions of prognostic factors, see Figure 6.

We define probability *P* to determine that the new subject is allocated to minimize delta with probability=*P* and to minimize D with probability=1–*P*, where *P* is chosen based on the original paper-proposed function: *P*=1–0.95^δ^.

## Results

All the functions in the system that we describe here have been fully tested in 2 popular Web browsers (ie, Internet Explorer 8.0 and Firefox) and already implemented in actual RCTs, 1 of which is a recently completed 3-arm study, Evaluation of Lifestyle Interventions to Treat Elevated Cardiometabolic Risk in Primary Care (E-LITE; ClinicalTrials.gov NCT00842426). E-LITE was designed to evaluate 2 behavioral weight-management interventions compared with usual care, in 1 primary care clinic of a large multispecialty group practice in Northern California [18]. The protocol specifies 7 prognostic factors for randomization: age, gender, race, pretrial online access to personal health records, fasting blood glucose, body mass index, and waist circumference. The Pocock-Simon minimization method was used. The summary table of all randomized records (n=241), which was generated by the Web-based system (with the exception of the *P* values), shows better than chance balance across all 7 prognostic factors among the 3 treatment arms (Table 1).

**Table 1:** Between-group differences in prognostic factors for the Evaluation of Lifestyle Interventions to Treat Elevated Cardiometabolic Risk in Primary Care (E-LITE) study.

Factor and level		Number in each treatment			Total	Max group difference	*P* value
		1	2	3			
**Age (years)**							
	18-44	21	19	20	60	2	.84
	45-64	52	46	48	146	6	
	65-79	8	13	12	33	5	
	80-100	0	1	1	2	1	
**Blood glucose (mg/dL)**							
	0-99	39	34	37	110	5	.41
	100-109	31	30	31	92	1	
	110-119	7	11	13	31	6	
	120-125	4	4	0	8	4	
**Body mass index (kg/m^2^)**							
	25-29.9	38	37	38	113	1	.99
	30-34.9	25	27	24	76	3	
	35-39.9	12	9	11	32	3	
	≥40	6	6	8	20	2	
**Gender**							
	Male	44	41	44	129	3	.94
	Female	37	38	37	112	1	
**Access to personal health records**							
	No	15	15	18	48	3	.81
	Yes	66	64	63	193	3	
**Race**							
	Hispanic	2	4	4	10	2	.93
	Asian/Pacific Islander	14	13	14	41	1	
	Non-Hispanic white	64	61	63	188	3	
	Other	1	1	0	2	1	
**Waist circumference (cm)**							
	<37	9	10	11	30	2	.99
	37-<40	23	19	21	63	4	
	40-<42	15	16	14	45	2	
	≥42	34	34	35	103	1	
Total		81	79	81	241	2	

## Discussion

### Principal Findings

We have developed a Web-based randomization system to facilitate use of the Pocock-Simon, symmetric KLD, and 2-way minimization methods. It provides user-friendly and error-resistant Web interfaces that are applicable to single- and double-blind trials as well as single-center and multicenter trials.

Randomization ensures that research subjects are assigned to a treatment independent of baseline characteristics, measured or unmeasured, including characteristics that are the current values of potential outcomes of interest. Minimization as an adaptive randomization procedure has the desirable features of minimizing accidental bias while maximizing the precision of treatment effect estimates, particularly in small trials [8,19]. Given that methods to improve the prospects for balance increase the risk of selection bias [20] and the nature of the trade-off depends on the details (eg, masking or not, knowledge or ignorance of baseline prognostic factors), the proper choice of biased assignment probability *P*_i_ specifically for Pocock-Simon and symmetric KLD methods varies according to individual study circumstances [2,21]. Our Web-based randomization system incorporates Efron’s biased-coin principle [3] and allows users to specify *P*_i_ when defining a new project and adjust it after project initiation if warranted (eg, if the initial *P*_i_ leads to imbalance measures exceeding a prespecified threshold in a given study).

Interactions between prognostic factors may affect response to treatment. It would be impractical to balance for all covariate interactions of any order in most clinical trials [2]. Nevertheless, all 3 minimization methods included in MinimRan can incorporate a first-order interaction between 2 categorical prognostic factors by creating a new variable whose levels correspond to all combinations of the 2 factors [2]. For example, a variable indicating gender (female, male) by smoking status (smoker, nonsmoker) interaction would have 4 levels: (1) female and smoker, (2) female and nonsmoker, (3) male and smoker, and (4) male and nonsmoker. Additionally, Endo et al’s symmetric KLD method [5] can also account for first-order interactions between a categorical and a continuous prognostic factor and between 2 continuous prognostic factors.

Decisions regarding appropriate *P*_i_ values specifically for Pocock-Simon and symmetric KLD methods and interaction terms between prognostic factors need to be study specific and should only be made by experienced researchers, preferably in consultation with a qualified biostatistician. The final selections should be clearly documented in the study protocol.

For a 2-arm study, the system provides the option of using the Pocock-Simon method [2], Endo et al’s symmetric KLD method [5], or the 2-way minimization method [4]. Although the KLD method has the advantage of permitting continuous and categorical prognostic factors and the 2-way minimization method protects unpredictability of new subject allocation, both algorithms are currently limited to randomization in 2-arm trials [4,5]. In contrast, the Pocock-Simon method can accommodate RCTs with more than 2 arms.

To support potential users of the MinimRan Web-based randomization system, we provide an online Q&A page and downloadable user manual, as well as a test-run option using dummy data (up to 10 subjects). In addition, we provide users with the option of contacting our development and super user team regarding tailoring characteristics of the program to their specific needs. For example, although the flexibility of our stand-alone system allows it to be used at institutions that do not yet have electronic data capture (EDC) or clinical trial management (CTM) systems, there may be users who want our randomization system to be integrated within their EDC and/or CTM systems, which are becoming more frequent in industry trial settings [22]. Similarly, academic and private or public health care centers that less frequently have EDC/CTM systems, but that use electronic health record (EHR) systems and also conduct clinical trials may seek our assistance to connect our Web-based randomization system to their EHR. There are administrative and regulatory requirements, however, that make such integration with the EHR challenging but not impossible [23]. For instance, integration requires institutional review board (IRB) authorization and strict adherence to the Health Insurance Portability and Accountability Act (HIPAA) privacy and security rules, which entail considerable effort. Furthermore, because many different EDC, CTM, and EHR systems exist, a Web-based stand-alone randomization system, such as the one described here, that is readily adaptable to different potential contexts of use has important practical value.

### Conclusion

The Web-based randomization system (MinimRan) described in this paper provides clinical trialists with a robust, flexible, and readily accessible tool for implementing covariate-adaptive biased-coin randomization. We have presented the system’s essential statistical and computer programming features and provided an example of the randomization results that it generated in 1 of our recent RCTs. A tool such as this can facilitate translation of validated randomization methods into broad, efficient use in clinical research.

## Multimedia Appendix 1

Technical details for the Web system and instruction for randomizing multi-site double-blind trial.

## Abbreviations

CSS: Cascading Style Sheets
CSV: comma-separated values
CTM: clinical trial management
EDC: electronic data capture
EHR: electronic health record
GUI: graphical user interfaces
JDBC: Java Database Connectivity
JSP: Java Server Page
KLD: Kullback–Leibler divergence
RCT: randomized controlled trial
