# Supplementary material for: An Easily Accessible Web-Based Minimization Random Allocation System for Clinical Trials
Source: J Med Internet Res. 2013 Jul 19;15(7):e139. doi: 10.2196/jmir.2392 (PMC3785994; doi:10.2196/jmir.2392)
Supplement: Supplementary file 1 [file jmir_v15i7e139_app1.pdf]

An easily accessed web-based minimization random allocation system for clinical trials

Table of Contents for Appendix

Definitions of single- and double- blind designs.. 2

Three-tier system architecture... 2

  Figure 1. Three-tier system architecture.. 3

Backend Database design... 3

  Figure 2. Database structure.. 5

Stepwise instruction for multi-site double-blind trial (e.g., drug trial)...................................6

Definitions of single- and double- blind designs

Single blind usually refers to either the subject or the researcher (i.e., an investigator or a staff person involved in the design, conduct, and/or reporting of a study) being unaware of the treatment assignment, whereas double blind usually refers to the subject and researcher are both unaware . In the case of single-blind studies where researchers need to be unblinded by design (e.g., in group-based behavioral intervention trials), it is recommended that only designated researchers on the study team be unblinded (e.g., the staff delivering the group intervention), while shielding the other researchers (e.g., outcome assessors, data analysts) to protect objectivity in data collection and outcome adjudication.

Three-tier system architecture

The system uses a three-tier architecture, which is the most widely used client-server architecture. The benefits of this technology are (1) easy access and user friendly interface; (2) ability to accommodate multiple projects; and (3) ability to manage and maintain each tier without affecting the other tiers. The three-tier architecture consists of a presentation tier, logic tier, and data tier (Fig. 1). The presentation tier is the user interface, which collects and displays information from the logic tier through a web browser. The logic tier is the middle-tier, which communicates with both the presentation and data tiers, provides logic processing and data access, and hides technical details from users. The logic tier uses a web server and an application server. Tomcat server was chosen as the web server because it is open, standard, robust, and low cost. We built the web application using Java application Java Server Page (JSP)

along with Cascading Style Sheets (CSS) and JavaScript. The data tier is the backend MySQL database server that stores and retrieves data, keeps data centralized and separate from the presentation and logic tiers, and improves scalability and performance. Finally, Java Database Connectivity (JDBC) achieves database-independent connectivity between the JAVA programming language and MySQL database. Both the Tomcat web server and MySQL sever are running on Microsoft windows Server 2003 Standard Edition. The system can be accessed using Internet Explorer 8.0 or higher at http://studies.pamfri.org:8080/minimRan/index.jsp.

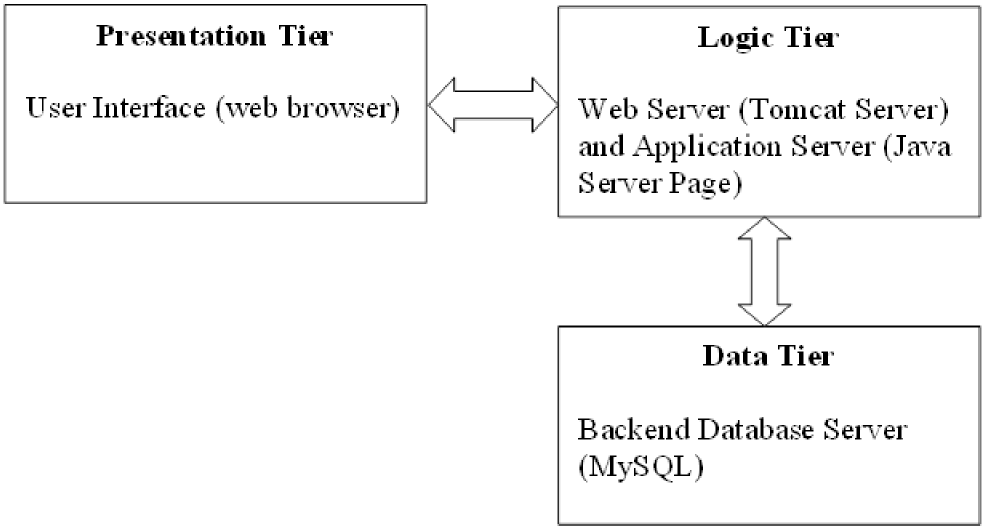

Figure 1. Three-tier system architecture.

Backend Database design

The relational database built for MinimRan makes the system dynamic, flexible, scalable, and reliable. It is easily understandable with the use of MySQL and integrates well with various data processing and analysis applications. The series of linked tables in the database make it easy to use to build, manage, and query data as well as to access, update, and search data across multiple tables. This structure also has the advantage of interfacing with many third-party tools such as the JSP, which our system uses.

The system generates eight tables for both single-blind and double-blind trials and one additional table for double-blinded trials only (Fig. 4). The Project, Factor, Level, and Prob tables store basic information entered during project initiation and definition stages, including study groups, study sites, prognostic factors (with logic rules if available), and biased assignment probability. The primary keys Project_ID in Table Project, Factor_ID in Table Factor, and Level_ID in Table Level, identify a unique project, the project's unique prognostic factors (which can be categorical or continuous), and a categorical factor's unique levels, respectively. Table Prob contains the user-defined biased assignment probabilities for each project (Project_ID) in Table Project. The Account, Project, and Account_Project tables are essential for project access privileges. Table Account stores users' login information. Data regarding all accessible projects (Project_ID) and privileges for individual users are kept in Table Account_Project. In every ongoing project, Table Subject Info is updated during each randomization run with the new subject's ID and values for the prognostic factors. Table Randomization records process data on all random allocations (e.g., coded group numbers, randomization sequence numbers, imbalance scores, randomization probabilities), which will permit quality assurance and replication. As described in section 2.4, for double-blind trials only, Table Masked_Num containing masked numbers and matching coded group numbers (GroupAssigned) is generated when the project is initiated and will be updated with subject IDs as randomization proceeds.

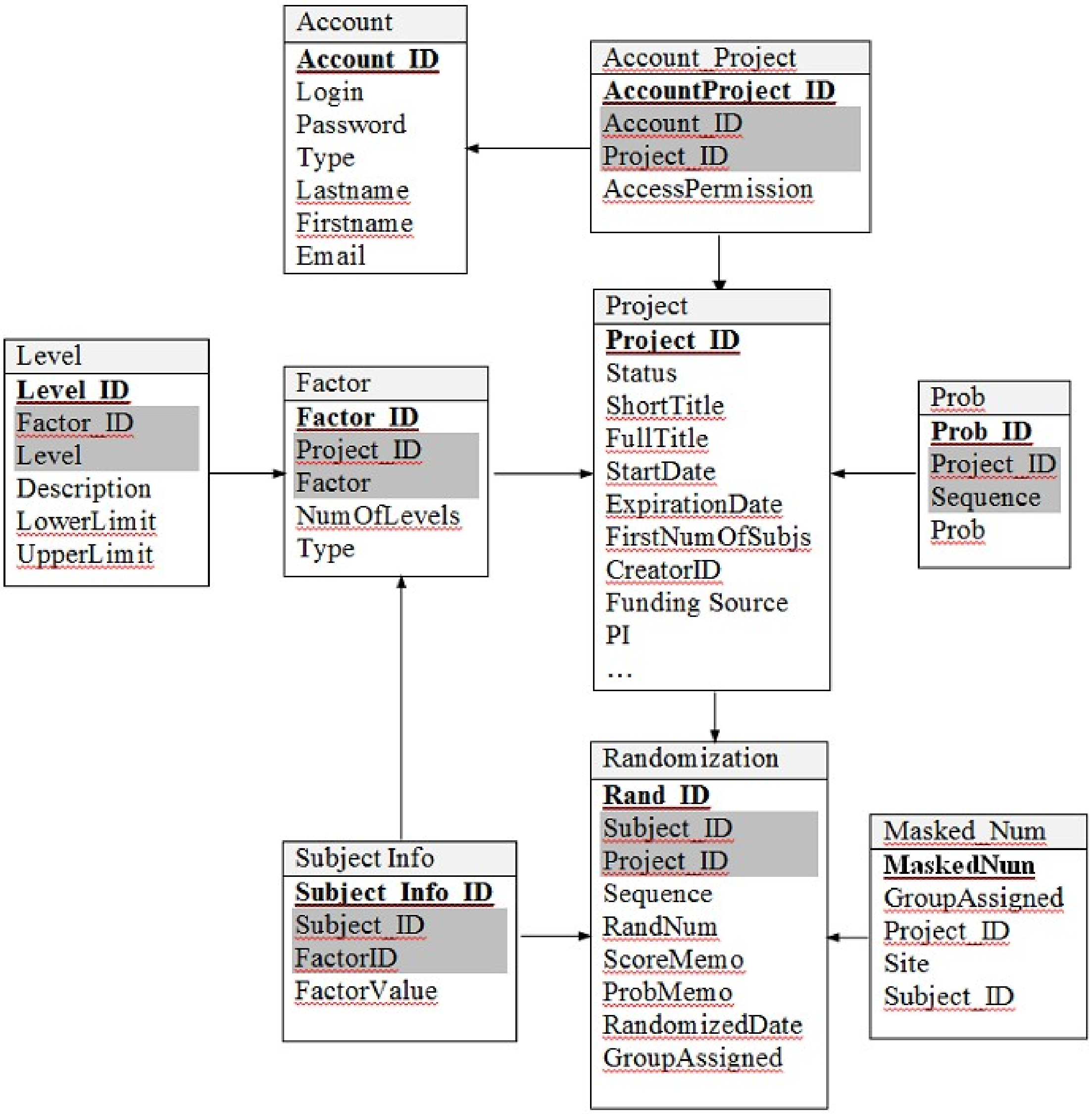

Figure 2. Database structure.

Note: Each table has a primary key (indicated with bold underlining), which uniquely identifies a record in the table. Where applicable, an alternative method of locating the same record is to use the “column” combinations highlighted in grey within the above boxes. The Masked_Num table is generated and updated for double-blind trials only.

Stepwise instruction for multi-site double-blind trial (e.g., drug trial)

Step 1: In the initial project set-up stage, project manager is required to input projected maximum number of subjects for each study site (required for double-blind trials only), as described on page 10 of the manuscript:

“Definition of study parameters by the authorized project manager. The parameters include single- or double-blind trial, number of study groups and group names (optional), number and short names of study sites, projected maximum number of subjects for each study site (required for double-blind trials only), minimization method selected (Pocock-Simon, symmetric KLD, or two-way minimization), biased assignment probability (not required for two-way minimization), prognostic factors, and levels of each categorical factor. ”

Step 2: Only the third party can access the table with masked numbers and matching coded group numbers or group names stratified by study site, as described on page 12:

“The system generates a Masked_Num table upon completion of the project initiation steps (section 2.4) and before randomization of the first subject in a double-blind trial. The table contains masked numbers and matching coded group numbers or group names (by study site if a multi-site trial), which only a designated third-party general user can access (section 2.3) and download (as a CSV file) for encoding the treatments (e.g., using masked numbers on drug bottle labels for distribution and tracking).”

Step 3: The third party on a double-blind trial will be responsible for matching the assigned masked numbers and the actual study groups, as described on page 12-13:

“The system provides project managers and general users performing randomization on double-blind trials with subjects’ assigned masked numbers but not the associated group numbers. The

user projected maximum number of subjects to be enrolled plus 10% more determines the number of masked numbers generated by the system. The system will generate additional masked numbers if 90% of the initial set of numbers for any of the study groups have been assigned. If the study includes multiple sites, the above assignments will apply for each site. A designated user on a single-blind trial who is involved in conducting the research and has permission to access randomization results and the third party on a double-blind trial will be responsible for matching the randomization results and the actual study groups."

References

[1] Department of Health and Human Services F. International conference on harmonisation; guidance on statistical principles for clinical trials; availability--FDA. Notice. Fed Regist. 1998;63:49583-98.

[2] Schulz KF, Grimes DA. Blinding in randomised trials: hiding who got what. Lancet. 2002;359:696-700.
